# The proactive health behavior and somatic functional status in Chinese rural chronic patients: the mediating effects of social participation

**DOI:** 10.3389/fpubh.2025.1668760

**Published:** 2025-09-10

**Authors:** Yangzhen Huang, Hua Qing, Yangyang Pan, Chunying Wang, Heng Dong, Jia Song, Kangkang Zhang, Yilin Wei, Shangfeng Tang, Min Zhang

**Affiliations:** ^1^School of Medicine and Health Management, Tongji Medical College of Huazhong University of Science and Technology, Wuhan, China; ^2^Research Center for Rural Health Service, Key Research Institute of Humanities and Social Sciences of Hubei Provincial Department of Education, Wuhan, China; ^3^Department of Neurology, Shanxi Bethune Hospital, Shanxi Academy of Medical Sciences, Third Hospital of Shanxi Medical University, Tongji Shanxi Hospital, Taiyuan, China

**Keywords:** proactive health behavior, social participation, somatic function status, rural health, chronic disease management

## Abstract

**Background:**

As chronic diseases become more prevalent in rural China, maintaining health and functional capacity has become a major challenge. In this context, individual proactive health behaviors (PHB) may play a crucial role. However, the mechanisms through which PHB influence somatic functional status (SFS) are still unclear. Particularly, the role of social participation (SP) in this process remains unexplored.

**Methods:**

A cross-sectional survey was conducted among 3,295 chronic disease patients in rural China. Data on PHB and SFS was evaluated using validated Likert scales. Data on SP were collected using a binary-response questionnaire covering seven domains of activity. Descriptive statistics were used to characterize the sample demographics and the distributions of key variables. Hierarchical regression analysis was performed to test the mediation effects.

**Results:**

The mean scores for participants were 24.98 ± 5.89 for PHB, 2.22 ± 1.21 for SP, and 43.33 ± 10.00 for SFS. Significant correlations were found between PHB and SP (*r* = 0.21, *p* < 0.001), SP and SFS (*r* = 0.23, *p* < 0.001), and PHB and SFS (*r* = 0.11, *p* < 0.001). Hierarchical regression and bootstrap analyses confirmed that SP partially mediated the relationship between PHB and SFS, explaining 42.01% of the total effect.

**Conclusion:**

Among rural patients with chronic diseases in China, individual health proactivity primarily enhances somatic function by promoting social participation. Therefore, integrated interventions that encourage both proactive health behaviors and social participation are recommended. These interventions can optimize chronic disease management outcomes in this population.

## 1 Introduction

The escalating burden of chronic diseases poses a formidable challenge to global health systems (1–3), particularly in rural area where aging populations and limited healthcare resources converge (4–6). As predicted, the prevalence of chronic noncommunicable diseases will increase to 40% by 2030 in China (7, 8). Despite nationwide initiatives like the “Healthy China 2030” strategy and the National Chronic Disease Comprehensive Prevention and Control Demonstration Zone Program (9), rural areas persistently demonstrate higher growth rates in chronic disease prevalence compared to urban counterparts (10–12). This disparity underscores an urgent need to identify modifiable behavioral factors that can optimize functional health outcomes within resource-constrained settings. Additionally, this study specifically focuses on patients with hypertension, diabetes, and stroke. These conditions were selected because they are the most prevalent chronic diseases in rural China and are major contributors to long-term disability and functional decline (13–15), making them a critical priority for public health intervention (16). According to the latest research data, the prevalence of hypertension in China is approximately 29.6% (17), diabetes is 13.7% (18), and stroke is 1.8% (19). Their high prevalence also ensures a sufficiently large and representative sample for our analysis.

Emerging paradigms in health promotion emphasize proactive health behavior (PHB) – a construct that represents an individual’s proactive engagement in managing their health, encompassing anticipatory health monitoring, voluntary participation in preventive care, and collaborative decision-making with healthcare providers (20, 21). PHB moves beyond passive disease management by empowering individuals to systematically acquire self-management skills through information-seeking and resource utilization (22, 23), aligning with the World Health Organization’s framework for patient-centered chronic care (24). While PHB is associated with better disease control (25), their direct impact on preserving somatic functional status (SFS)—the ability to perform daily physical activities—may be limited, especially in complex rural settings (26, 27). This is because the translation of health behaviors into tangible functional benefits often depends on psychological and social mechanisms, such as enhanced self-efficacy and social support (28, 29). Therefore, the pathway from individual agency to functional health is likely mediated by broader social processes (30, 31).

To understand these processes, the social-ecological model provides a robust theoretical framework, positing that health outcomes are shaped by the interplay between individual behaviors and multiple levels of the social environment (32, 33). Within this framework, social participation (SP) is one of the critical social determinant of health, which means active involvement in community and social activities (34), influencing both mental and physical well-being (35, 36). It is important to distinguish the concept of social participation used in this study from social prescribing, a formal healthcare intervention where healthcare professionals refer patients to non-clinical community services via a link worker (37, 38). Our measure of social participation captures the individual’s spontaneous and voluntary engagement in community activities, reflecting their inherent level of social integration (39, 40). According to the social-ecological model, individual-level factors and community-level factors are not isolated; rather, they interact in a reciprocal manner (41, 42). For instance, an individual’s health proactivity may empower them to overcome barriers and engage more confidently in social life (43), and a supportive social environment can facilitate access to health resources and reinforce healthy behaviors (44). Despite the theoretical plausibility of this interaction, several critical knowledge gaps still exists. First, the interaction between individual-level PHB and community-level SP in influencing health has not been fully examined. Second, few studies have explored SP as a potential mediator between PHB and SFS. Given the latent complex relationship between PHB and SFS, exploring only their direct effects would undoubtedly oversimplify the issue. Therefore, it is essential to understand how key factors like SP mediate the relationship between PHB and SFS, offering more actionable insights for clinical practice.

This research aims to clarify the associations among PHB, SP, and SFS, and to quantify the role of SP as a pathway through which health-proactive behaviors contribute to functional preservation. The analysis is framed within the unique sociocultural and healthcare context of rural China. The findings are expected to provide insights for developing integrated public health strategies that utilize social participation to enhance the somatic functional well-being of rural populations.

## 2 Methods

### 2.1 Study design

#### 2.1.1 Participants and setting

The study was conducted from July to August 2023 using a stratified random sampling method. In the first stage, six townships in Qianjiang City, Hubei Province were selected as primary sampling units. The selected townships are representative of typical rural areas in central China, characterized by agrarian livelihoods, close-knit social networks, and a significant proportion of the population engaged in farming. In the second stage, individual patients were selected from chronic disease registries maintained by local health centers in each township, using systematic random sampling. The final sample included 3,600 participants, with a target allocation of 240 hypertension patients, 240 diabetes patients, and 120 stroke patients per township. This resulted in a total of 1,440 hypertension patients, 1,440 diabetes patients, and 720 stroke patients. For cases of multimorbidity, the first-reported condition was designated as the primary diagnosis to assign each participant to a single disease category for analysis. The numerical allocation aimed to balance the representation of the three target diseases, with larger sample sizes for hypertension and diabetes due to their higher prevalence in the target population.

#### 2.1.2 Data collection procedures

Data were collected through face-to-face interviews, with trained research staff directly entering the participants’ responses into the questionnaire on mobile devices via the Chinese online survey platform Wenjuanxing (wjx.cn). This approach ensured the survey was accessible to all eligible participants, particularly those who were illiterate or less familiar with digital technology. To ensure data quality and response validity, individuals with incomplete responses or unreasonably short completion times (<400 s, validated through pilot testing) were excluded, resulting in a final analytical sample of 3,295 participants.

#### 2.1.3 Ethical considerations

The study was approved by the Ethical Review Committee of Tongji Medical College, Huazhong University of Science and Technology ([2022] IEC-A251) (China). All participants or their legal guardians/appropriate representatives (in case of illiterate participants) provided informed consent to participate in this study.

### 2.2 Measurement of proactive health behavior

The proactive health behavior (PHB) scale was adapted from a validated instrument originally developed for Chinese hypertensive patients (45), demonstrating good validity and reliability in populations with chronic diseases. To focus specifically on the core construct of initiative and self-management in chronic disease care, we selected eight core items from the “proactive health responsibility” subdomain of this scale. The selected items were: (1) initiating physical examinations, (2) reporting physical discomfort, (3) participating in health education programs, (4) seeking health guidance, (5) obtaining multidisciplinary care advice, (6) consulting healthcare professionals for health recommendations, (7) clarifying medical doubts proactively, and (8) acquiring disease self-management knowledge. Responses were captured using a 5-point Likert scale (1 = never to 5 = always), with total scores ranging from 8 to 40. The scale showed excellent internal consistency in our sample (Cronbach’s α = 0.810). To further assess its reliability within each disease subgroup, we calculated Cronbach’s alpha separately for patients with hypertension (Cronbach’s α = 0.805), diabetes (Cronbach’s α = 0.806), and stroke (Cronbach’s α = 0.814). These results demonstrated high reliability and internal consistency across all three chronic disease groups, confirming the scale’s applicability for use in this mixed cohort.

### 2.3 Measurement of somatic functional status

Somatic functional status (SFS) was assessed using the validated Functional Activities Questionnaire (FAQ), a widely recognized and reliable tool for measuring difficulties in activities of daily living (46–49). Respondents rated their capacity to perform essential daily tasks including meal preparation, financial management, and community mobility on a 5-point Likert scale, where 1 = “totally dependent on others” and 5 = “fully independent.” Total scores ranged from 10 to 50. The Chinese adaptation showed the strong internal consistency in our sample (Cronbach’s α = 0.95).

### 2.4 Measurement of social participation

Social participation, a complex and multidimensional concept reflecting individual engagement in community activities, was measured using a binary response (yes/no) questionnaire consisting of seven dimensions (50). The domains included political participation (civic activities including voting and community decision-making), economic participation (income-generating activities like formal employment or agricultural work), voluntary participation (unpaid community services like environmental clean-up initiatives), family participation (e.g., caregiving and intergenerational support), community participation (e.g., membership in local organizations), digital participation (e.g., online social interaction), and other forms of participation. The total score of the scale was the sum of the dimension scores, ranging from 0 to 7. The scale showed low internal consistency (Cronbach’s α = 0.149). This is expected, as the scale aims to capture the broad scope of engagement across seven diverse domains, rather than a single, unified construct. Thus, it reflects the distinct nature of these activities.

### 2.5 Measurement of covariates

To control for confounding demographic factors, we selected several covariates based on literature review and included them in the regression model (51, 52). These variables were categorized and incorporated as follows: (1) gender (male, female); (2) age (59 and below, 60–69, and 70 and above); (3) education level (uneducated, primary, middle, high school, and above); (4) occupation type (unemployed, farmers, freelancers, administrators, private owners, and others); (5) household income (less than ¥500 per month, ¥500–1,000 per month, ¥1,000–1,500 per month, ¥1,500–2,000 per month, more than ¥2,000 per month, and not disclosed).

### 2.6 Analytical strategy

The categorical variables were described using frequency (%), and the differences in frequency were examined using $\chi^{2}$ test. Pearson correlation was used to analyze the correlation between main variables. Hierarchical regression analysis (53) was used to examine the mediating effect of SP (M) on the relationship between PHB (X) and SFS (Y). During model construction, the following tests were conducted: (1) examining whether PHB (X) significantly affects SP(M); (2) testing whether PHB(X) significantly influences SFS(Y); and (3) assessing whether SP(M) significantly impacts SFS(Y). If these tests were validated, the next step was to investigate whether the effect of PHB(X) on SFS(Y) weakened upon the inclusion of SP(M), and whether SP(M) still significantly affected SFS(Y). If the results were affirmative, it would indicate the presence of a mediating effect. Subsequently, a bootstrap analysis with 5,000 resamples was used to test the significance of the mediation effect. All analyses were conducted with SPSS 27.0, with a *p*-value < 0.05 considered as statistically significant.

## 3 Results

### 3.1 Participant demographics

This study included 3,295 patients with chronic diseases. The majority had hypertension (*n* = 1,270, 38.5%) or diabetes (*n* = 1,285, 39.0%), followed by stroke (*n* = 664, 20.2%). A small proportion of participants (*n* = 76, 2.3%) reported having multimorbidity, defined as the presence of two or more chronic conditions. SFS scores differed significantly across all demographic and socioeconomic groups (all *p* < 0.001). Detailed demographic characteristics and comparisons of SFS levels across different demographic groups are presented in Table 1.

**Table 1:** Demographic characteristics of the sample (*N* = 3,295).

Characteristics	Participants [*N* (%)]	Somatic functional status				
		Bad	Medium	Good	χ ^2^	*P* value
Gender						
Male	1,698 (51.5)	129 (44.9)	208 (42.3)	1,361 (54.1)	28.467	<0.001
Female	1,597 (48.5)	158 (55.1)	284 (57.7)	1,155 (45.9)		
Age						
<59	962 (29.2)	34 (11.8)	52 (10.6)	876 (34.8)	364.259	<0.001
60–69	1,295 (39.3)	68 (23.7)	177 (36.0)	1,050 (41.7)		
>70	1,038 (31.5)	185 (64.5)	263 (53.5)	590 (23.4)		
NCDs type						
Hypertension	1,270 (38.5)	34 (11.8)	157 (31.9)	1,079 (42.9)	601.348	<0.001
Diabetes	1,285 (39.0)	42 (14.6)	157 (31.9)	1,086 (43.2)		
Stroke	664 (20.2)	194 (67.6)	168 (34.1)	302 (12.0)		
Multimorbidity	76 (2.3)	17 (5.9)	10 (2.0)	49 (1.9)		
Education level						
Uneducated	731 (22.2)	114 (39.7)	172 (35.0)	445 (17.7)	190.109	<0.001
Primary school	1,250 (37.9)	112 (39.0)	211 (42.9)	927 (36.8)		
Middle school	1,027 (31.2)	53 (18.5)	92 (18.7)	882 (35.1)		
High school and above	287 (8.7)	8 (2.8)	17 (3.5)	262 (10.4)		
Occupation type						
Unemployed	893 (27.1)	132 (46.0)	168 (34.1)	593 (23.6)	107.599	<0.001
Farmers	1,975 (59.9)	132 (46.0)	281 (57.1)	1,562 (62.1)		
Freelancers	183 (5.6)	3 (1.0)	12 (2.4)	168 (6.7)		
Administrative workers	73 (2.2)	5 (1.7)	4 (0.8)	64 (2.5)		
Private owners	24 (0.7)	0 (0.0)	1 (0.2)	23 (0.9)		
Others	147 (4.5)	15 (5.2)	26 (5.3)	106 (4.2)		
Household income						
<500	890 (27.0)	145 (50.5)	197 (40.0)	548 (21.8)	215.380	<0.001
500–1,000	591 (17.9)	49 (17.1)	105 (21.3)	437 (17.4)		
1,000–1,500	398 (12.1)	12 (4.2)	50 (10.2)	336 (13.4)		
1,500–2,000	325 (9.9)	15 (5.2)	28 (5.7)	282 (11.2)		
>2,000	904 (27.4)	51 (17.8)	72 (14.6)	781 (31.0)		
Non-disclosure	187 (5.7)	15 (5.2)	40 (8.1)	132 (5.2)		

### 3.2 Correlation analysis among PHB, SP, and SFS

The mean scores were 24.98 ± 5.89 for proactive health behavior, 2.22 ± 1.21 for social participation, and 43.33 ± 10.00 for somatic functional status. In the correlation analysis, demographic factors such as gender, age, educational level occupation type, and per capita monthly household income were meticulously controlled. The derived findings from Pearson’s correlation analysis conspicuously indicated a statistically significant, positive correlation between the measure of social participation and both the proactive health behavior (*r* = 0.21, *p* < 0.001) and the somatic functional status (*r* = 0.23, *p* < 0.001). Furthermore, a significant positive association was also observed between the proactive health behavior and the somatic functional status (*r* = 0.11, *p* < 0.001), as detailed in Table 2.

**Table 2:** Correlations between PHB, SFS, and SP.

	Mean	Standard Deviation	SP	PHB	SFS
SP	2.22	1.21	1.00		
PHB	24.98	5.90	0.21***	1.00	
SFS	43.33	10.00	0.23***	0.11***	1.00

PHB, proactive health behavior; SFS, somatic functional status; SP, social participation, *p < 0.05, **p < 0.01, ***p < 0.001.

### 3.3 Mediating role of SP between PHB and SFS

**Figure 1:**
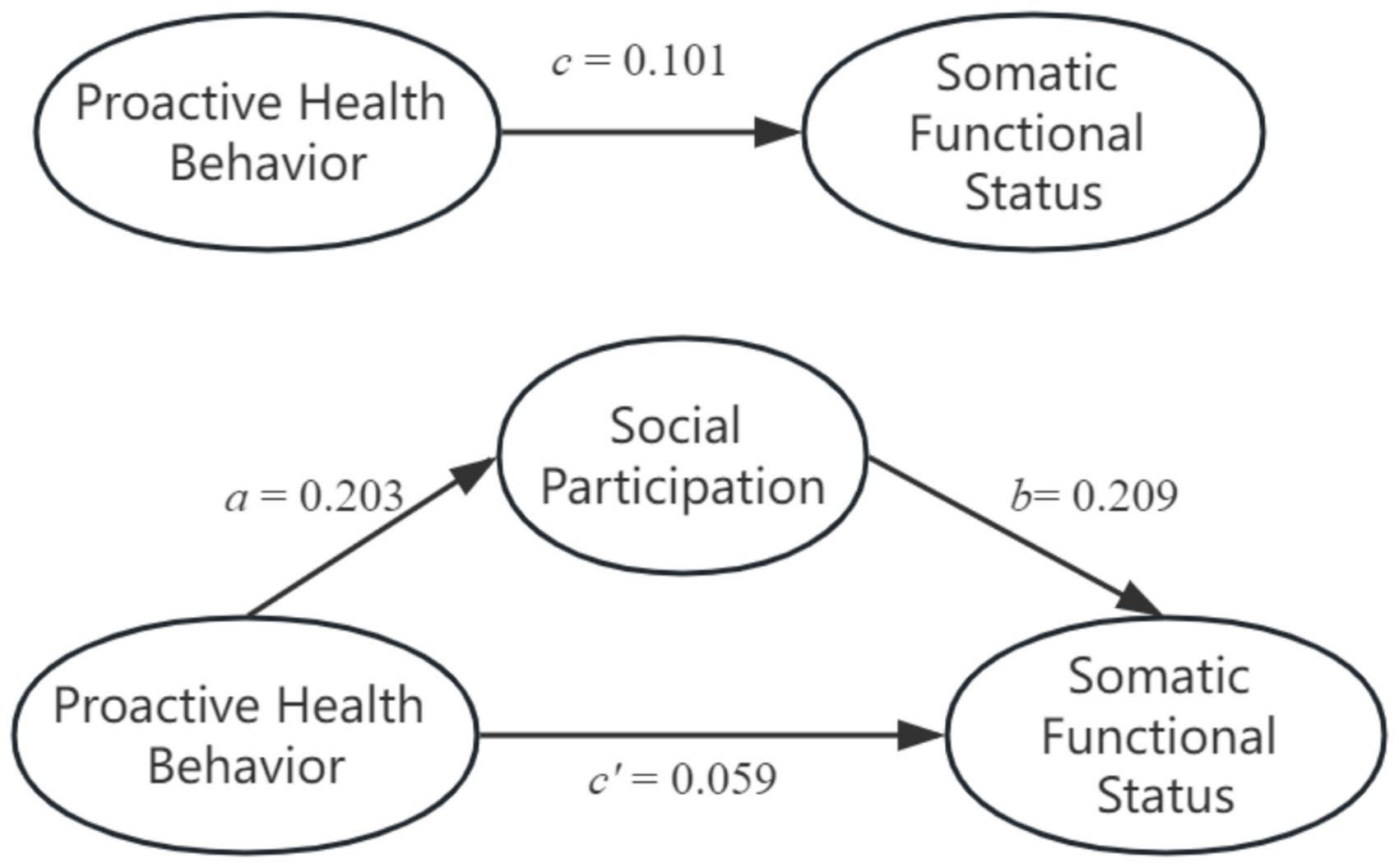
The mediating effect of social participation between proactive health behavior and somatic functional status.

In the analysis presented within Table 3, Model 1 elucidated that, among the plethora of covariates examined, only age (β *=* −0.182, *p* < 0.001) and education levels (β *=* 0.084, *p* < 0.001) exhibited significant influence on the levels of social participation among rural chronic disease patients. Model 2 established a substantial positive effect of proactive health behavior on social participation (β = 0.203, *p* < 0.001). Furthermore, upon adjustment for the influence of extraneous variables, Model 4 revealed a noteworthy positive correlation between proactive health behavior and the somatic functional status of the patients (β = 0.101, *p* < 0.001). Additionally, the exploration extended to the between proactive health behavior, social participation, and the somatic functional status of patients, with findings encapsulated in Model 5. Herein, it was demonstrated that social participation acted as a mediating factor between proactive health behavior and the somatic functional status (β = 0.209, *p* < 0.001). Among them, the mediation effect (ab) was 0.042, the total effect (c) was 0.101, and 42.01% of the total effect was realized through the mediator of social participation, as illustrated in Figure 1. To confirm the significance of the indirect effect, we conducted a bootstrap analysis. The mediating effect was found to be statistically significant, as the 95% confidence interval did not include zero. This result strongly supports the role of SP as a significant mediator in the relationship between PHB and SFS.

**Table 3:** Results of mediating effects analysis.

Variables	Social participation		Somatic functional status		
	Model 1	Model 2	Model 3	Model 4	Model 5
Constant	3.450	2.460	58.688	54.585	50.326
Covariates					
Gender	−0.008	−0.012	−0.014	−0.016	−0.014
Age	−0.182***	−0.180***	−0.286***	−0.285***	−0.247***
Education level	0.084***	0.063**	0.107***	0.097***	0.084***
Occupation type	0.016	0.027	0.020	0.025	0.019
Household income	0.028	0.022	0.121***	0.118***	0.114***
Independent variable					
Proactive health behavior		0.203***		0.101***	0.059***
Mediating variable					
Social participation					0.209***
*R* ^2^	0.058	0.099	0.158	0.168	0.206
*∆R* ^2^	0.058	0.041	0.158	0.010	0.039
*F*	40.560***	60.000***	123.105***	110.509***	122.763***
*∆F*		148.127***		40.196***	163.516***

*p < 0.05, **p < 0.01, ***p < 0.001. The regression coefficients in the table are standardized.

## 4 Discussion

This study offers new insights into the relationship between individual agency, social context, and physical functional health in the management of chronic diseases in rural areas. The modest direct correlation between PHB and SFS (*r* = 0.11), coupled with a substantial 42.01% mediation effect of SP, suggests that PHB may not lead to immediate functional improvements. Instead, they promote increased SP, which creates a supportive environment for functional preservation. The functional benefits of individual health initiatives are largely realized through social integration within the community. Moreover, younger age and higher education levels are significantly associated with better somatic functional status, underscoring the influence of sociodemographic factors on functional health outcomes.

The core finding of a significant mediation effect can be explained by the interplay of behavioral mechanisms and the unique socio-cultural context of rural China. Mechanistically, PHB likely enhances patients’ health literacy and self-efficacy (54). This, in turn, empowers them to participate more confidently and purposefully in social activities. The resulting increase in SP fosters a “health-enabling social ecology,” which provides platforms for both formal and informal peer support, health information exchange, and collective monitoring (55). At the same time, SP helps reduce psychological stress and boosts self-efficacy (56, 57), all of which contribute directly to the preservation of physical function. The remarkably high mediation proportion is particularly reflective of the rural Chinese context. Rural area often function as “acquaintance society,” characterized by strong collectivist values and dense social networks (58, 59). In this environment, SP is not merely a leisure activity, it also serves as a fundamental expression of social identity and responsibility. The rich social capital embedded in these areas enables SP to significantly amplify the benefits of PHB, transforming individual health efforts into collective well-being. This explains why a large portion of PHB’s impact on SFS is mediated through social participation.

Our results align with and extend prior research in three aspects. First, prior studies on rural populations have primarily focused on passive disease management strategies (60, 61). In contrast, this study shifts the emphasis to individual-level PHB among patients with chronic conditions. The moderate levels of PHB observed in our study (*M* = 24.98 ± 5.90) highlight considerable potential for improvement. This suggests that there is significant room for proactive behavioral interventions (62–64). Second, the mediating role of SP (β = 0.209, *p* < 0.001) corroborates ecological models of health (65), revealing how individual health behaviors interact with community-level social capital. This finding challenges conventional wisdom that rural health outcomes primarily depend on healthcare infrastructure (66, 67), instead highlighting the synergistic potential of combining PHB promotion with community engagement programs. Third, the age gradient in SP (β = −0.182, *p* < 0.001) underscores the critical need for age-tailored health communication strategies, particularly as China’s rural population rapidly ages (68).

The findings of this study offers meaningful implications for theory and practice. Theoretically, the validated “PHB → SP → SFS” pathway enhances our understanding of how PHB may lead to positive health outcomes, emphasizing the crucial mediating role of SP. Practically, the results indicate that interventions focused solely on promoting PHB may have limited effectiveness. Instead, the impact of such interventions is likely to be more substantial when combined with efforts to foster SP. These findings advocate for a shift from isolated health education to integrated interventions that promote both PHB and SP, with a particular emphasis on counteracting the age-related decline in social participation. To implement this in rural settings, we recommend integrating health initiatives into existing social structures. For example, establishing “Health Corners” within popular gatherings like “(square dancing), “forming “Village Health Promotion Teams” led by trusted community leaders, and implementing a “Health Points” system that rewards both health actions and social participation. These context-specific strategies capitalize on the social capital inherent in rural areas, transforming individual health efforts into collective benefits. This approach offers a practical pathway for policies, such as “Healthy Villages,” to improve chronic disease management.

This study has several limitations. First, the cross-sectional design limits the ability to draw definitive causal conclusions, as reverse causation cannot be ruled out. Additionally, the use of self-reported data for all key variables may introduce social desirability bias. The sample was also drawn from a specific rural region in Hubei Province, which may limit the generalizability of the findings to other regions or cultural contexts. Furthermore, the partial mediation effect suggests that a significant portion of the variance in the PHB-SFS relationship remains unexplained. This implies that other factors, such as biological mechanisms or healthcare system elements, may play a role (69, 70). Future research should address these limitations by adopting longitudinal or intervention designs, incorporating objective biomarkers, and conducting multi-center studies to establish causal relationships and test the robustness of our mediation model across diverse populations.

## 5 Conclusion

This study provides empirical evidence that SP plays a significant role in mediating the relationship between PHB and SFS among chronic disease patients in rural China. Our results show that the functional benefits of health proactivity are primarily realized through SP, which accounts for 42.01% of the total effect. This highlights the importance of individual agency in health management, which is most effectively translated into preserved physical function when supported by a robust social environment. We recommend that public health strategies, especially within the framework of China’s “Healthy Villages” initiative, should combine the promotion of PHB with efforts to strengthen SP. This approach would leverage the synergistic effects of both factors on functional health. Such integration can be achieved by incorporating health promotion into existing village structures and social activities, utilizing the rich social capital found in rural life.

## Data Availability

The data analyzed in this study is subject to the following licenses/restrictions: The datasets are not publicly available to protect participant privacy but can be provided by the corresponding author upon reasonable request. Requests to access these datasets should be directed to sftang2018@hust.edu.cn.
